# Recent Advances in Ophthalmic Science

**Published:** 1866-05

**Authors:** 


*Recent Advances in Ophthalmic Science. The Boylston Prize Essay for* 1865. By HENRY W. WILLIAMS, M. D., Ophthalmic Surgeon to the City Hospital, Boston; University Lecturer on Ophthalmic Surgery in Harvard University; Member of the American Ophthalmological Society, etc., etc., etc. Boston: Ticknor & Fields. 1866.

The immense progress which has been made in Ophthalmic Surgery, within the past few years, makes a brief *resume* of the many brilliant advances in this department of medicine, a desideratum in the profession. It is more particularly the student of medicine and general practitioner, who have so much felt the urgent and growing necessity of a work which, without being too elaborate, would present all new facts in regard to the improved means of diagnosis, pathology and treatment of diseases of the eye. This little work of 166 pages supplies the demand, and is all that could be desired.

The first twenty-five pages are devoted to the Ophthalmoscope, its practical application, and the advantages to be derived from its use—illustrated with plates which furnish to the novice in these studies the most perfect demonstration of the principles involved and their application, that we recollect ever to have noticed in any previous work. To the Ophthalmoscope the author attributes many of the recent improvements—regarding its application as a means of diagnosis, the commencement of a new era in Ophthalmic Surgery. In speaking of this instrument the author says:

"It is not claiming too much to assert that the ophthalmoscope has done more to increase our knowledge of diseases of the eye than had been accomplished during a century by all other means. The practitioner is not now obliged to include a large number of deep-seated diseases of the eye under the designation 'Amaurosis,' to which the well-known remark of Walther was, unfortunately, but too apropos—'a condition where the patient sees nothing, and the doctor, also—nothing.' '*Je ner zustand, bei welchem der Kranke nichts sieht, aber der Arzt auch nichts.*' He is relieved from many embarrassing uncertainties in diagnosis, painful to himself, and more or less detrimental to patient, and is no longer in the dark; but, in regard to the interior of the eye, can speak of what he knows and testify to what he sees. As accurate diagnosis is and must be the basis of all successful treatment, the occulist can already point to brilliant therapeutic triumphs over diseases hitherto deemed incurable, which have directly resulted from the knowledge acquired by means of this instrument."

Considerable space is devoted to the modifications and improvements in the operation for cataract; several are discussed—one of which we have never seen suggested before. This method consists in the insertion of a suture in the cornea after the extraction of a cataract. The author says: "So far as I know, the insertion of sutures in the cornea, after extraction of the lens, to facilitate primary union of the flap, has never been performed or suggested, until lately by myself. I have done this in a considerable number of instances, and thus far with invariable success; and after careful observation of the results obtained, can advocate this method as possessing numerous and important advantages. I prefer a straight needle, only a quarter of an inch long, made by cutting off the requisite length from the head of the finest sewing needles and forming a new point. The needle is held and passed through the cornea by means of a pair of firm forceps, and the suture, formed by a single strand of silk, or the finest thread, is tied—not too tightly. This is allowed to remain until it cuts itself out, which is sometimes not for several days, or even weeks, as there is danger of re-opening the wound if its removal is attempted, unless during anæsthesia. From the usual intolerance of the cornea of the presence of foreign bodies, we might expect that the suture would give rise to much irritation, but such has not been the fact, as I have known it to remain in situ for seven weeks without causing inconvenience.

"Every one will appreciate the immense advantages to be gained by such a means of insuring immediate union of the corneal wound, if the question of tolerance be once determined in its favor."

If we had sufficient space, there are many other features of this interesting little volume that we would be pleased to notice. No work upon this subject contains so much valuable information in so small a compass. It certainly deserves a place in the library of every intelligent reading physician.

